# Characterization of Envira Fibers Endemic to the Amazon Rainforest and Their Potential for Reinforcement in Polymer Composites

**DOI:** 10.3390/polym17172284

**Published:** 2025-08-23

**Authors:** Miriane Alexandrino Pinheiro, Leoncio Soares Galvao Neto, Alisson Clay Rios da Silva, Sérgio Neves Monteiro, Felipe Perisse Duarte Lopes, Marcos Allan Leite dos Reis, Verônica Scarpini Candido

**Affiliations:** 1 Engineering of Natural Resources of the Amazon Program, Federal University of Pará, Ananindeua 67030-007, PA, Brazil; mirialexandrino@gmail.com (M.A.P.); leoleo.eu@gmail.com (L.S.G.N.); marcosallan@ufpa.br (M.A.L.d.R.); 2 Materials Science and Engineering Program, Federal University of Pará, Ananindeua 67030-007, PA, Brazil; alissonrios@ufpa.br; 3 Department of Mechanical and Materials Engineering, Military Institute of Engineering, Rio de Janeiro 22290-270, RJ, Brazil; snevesmonteiro@gmail.com; 4 Materials Science Program, North Fluminense State University-UENF, Campos dos Goytacazes 28013-602, RJ, Brazil; perisse@uenf.br

**Keywords:** envira fiber, natural fiber composites, characterization

## Abstract

Natural lignocellulosic fibers (NLFs) replacing synthetic fibers have been used as reinforcement in polymer matrix composites. In this work, a lesser-known NLF endemic to the Amazon region, the envira fiber (*Bocageopsis multiflora*), was analyzed for its basic physical, thermochemical, morphological, and mechanical characteristics. In addition, epoxy matrix composites with 10, 20, 30, and 40 vol% of continuous and aligned envira fibers were evaluated by Fourier transform infrared spectroscopy (FTIR) and tensile tests. The results were statistically compared by ANOVA and Tukey’s test. The density found for the envira fiber was 0.23 g/cm^3^. The crystallinity index and microfibrilar angle obtained were 69.5% and 7.07°, respectively. Fiber thermal stability was found up to around 210 °C. FTIR confirmed the presence of functional groups characteristic of NLFs. Morphological analysis by SEM revealed that the envira fiber displayed fine bundles of fibrils and a rough surface along its length. The average strength value of the envira fiber was found to be 62 MPa. FTIR analysis of the composites confirmed the presence of the main constituents of the epoxy resin and NLFs. The tensile strength results indicated that the envira fiber addition increased the strength of the composites up to 40 vol%. The analysis of the fracture region revealed brittle aspects. These results indicate that envira fibers present potential reinforcement for polymer matrix composites and can be used in engineering applications, favored by their lightness and cost-effectiveness.

## 1. Introduction

With technological and industrial advances, it has become increasingly necessary to develop engineering materials that have improved mechanical properties, such as high strength and rigidity, combined with ease of processing and low weight [1,2,3,4,5]. Fiber-reinforced polymer matrix composites are examples of materials with this combination. The most commonly used reinforced composites are produced with synthetic fibers. These composites are currently used in the aerospace, defense, automotive, sports, and wind energy industries [4,5,6,7,8,9,10]. However, composites made with synthetic fibers are relatively expensive, heavy, difficult to manufacture, require a large amount of energy to produce, and have a negative impact on human health and the environment [9,10,11,12,13,14,15,16].

The search for lightweight materials, especially in the automotive sector, has long been fueled by the quest for greater fuel efficiency and a reduction in greenhouse gas emissions [15,16,17]. Moreover, a concern for the environment has grown steadily in recent decades. This has led the scientific community to study possible materials that could replace their synthetic counterparts [16,17,18,19,20,21,22]. A promising way to replace or reduce the use of synthetic materials is to consider natural reinforcements in the production of composites. Natural fibers, especially fibers of plant origin, also known as natural lignocellulosic fibers (NLFs), have been increasingly investigated as possible reinforcements in composite materials [10,11,12,13,14,15,16,17,18,19,20,21,22,23,24,25].

Composite materials with NLF reinforcements have shown worldwide interest due to the demand for environmentally friendly materials combined with technological and economic viability [26,27,28,29]. These materials are lightweight and provide greater strength at a reasonable cost compared to other composites [6,9,18]. The reduction in abrasiveness facilitates the processing of NLFs and increases their recyclability [17,18,21]. Although synthetic fiber composites have a higher specific strength than NLF composites, the abundance of availability, low cost, and low density are factors that greatly influence the production of these materials [23,24,25]. The combination of these characteristics positions NLF-reinforced composites as an attractive competitor for applications in various industries, such as the automotive industry, where the demand for sustainable, high performance materials is paramount [17].

NLFs from numerous species have been investigated worldwide for use as reinforcements in composites. Just mentioning a few recent epoxy composite examples. Helaili et al. [30] studied the mechanical properties of alpha natural fiber composites in epoxy composites; with short and randomly oriented fibers, an increase in modulus and tensile strength was identified. Belouadah et al. [31] produced an epoxy resin-based composite reinforced with *Lygeum spartum* fibers; an improvement in tensile and flexural mechanical properties was observed compared to pure epoxy resin, as well as good fiber-matrix adhesion. Rekha et al. [32] evaluated the macroscopic characteristics of the surface of the bamboo fiber epoxy polymer matrix composite; the results indicated that the two factors that most influence the mechanical properties are length and ideal fiber content. Maguteeswaran et al. [33] studied the effect of alkaline treatment on the fiber extracted from the stem of the *Lakaran acacia* tree in an epoxy matrix. It was observed that the treatment improved the mechanical properties in tensile, flexural, and impact, improving the bond between the fiber and the epoxy matrix, which resulted in higher mechanical properties in the manufactured composite. Cotton fibers in epoxy [34] were evaluated for their mechanical, wear, and thermal properties. The authors observed that the composite showed good resistance with a value of 52 MPa, making it suitable for applications requiring robust mechanical properties. Owen et al. [35] produced and mechanically characterized water hyacinth fiber composites in epoxy; the authors observed a strong fiber/matrix interfacial bond between the treated fibers and the epoxy matrix, demonstrating that water hyacinth fibers have potential as an alternative material to replace synthetic fibers in composite applications. Studies with *Tectona grandis* fiber [36] showed that fiber reinforcement caused a significant improvement in the mechanical aspect of the composite. According to the authors, these results indicate that the material produced represents a viable substitute for synthetic reinforcements.

The Amazon rainforest has the greatest biodiversity on the planet, sheltering a wide variety of plant species, being the largest preserved forest in the world, and sheltering a vast number of NLF species that have the potential to be used as reinforcements in polymer matrices [25,26,27]. Oliveira et al. [25] studied tucum fiber (*Astrocaryum vulgare*) and concluded that composites might be applied as a substitute for conventional composites reinforced with synthetic fibers, particularly in multilayered armor for body protection. Marchi et al. [26] studied the characteristics of ubim fiber (*Geonoma baculifera*) and concluded that, due to its low density, approximately 0.73 g/cm^3^, its use as a reinforcing agent in polymer matrices enables the production of lighter composites with applications in civil construction. A study on the incorporation of caranan fibers (*Mauritiella armata)* as reinforcement in epoxy matrix composites showed that caranan fiber significantly increases the tensile strength of composites and can replace glass fibers [27]. Maciel et al. [37] performed a comparative study of composites reinforced with curaua (*Ananas erectifolius*) and glass fibers, concluding that, from the point of view of mechanical strength, curaua fibers can replace glass fibers. Additionally, composites with curaua fiber can show a weight reduction of up to 15% when compared to materials reinforced with glass fibers. The authors indicated that composites can be used in sports equipment, reducing both the weight and cost of the produced material. Mallow (*Urena lobata*) and tucum fibers were studied as reinforcements for polymeric biomatrices [38]. The results indicated that the composites prepared with a high content of lignocellulosic fibers showed promising performance in the mechanical tests applied, with excellent potential for replacing engineering materials. Studies carried out on buriti fiber (*Mauritia flexuosa*) [39] indicated a value of 58% for cellulose content, indicating that this fiber has good stiffness and strength. Lopes Jr. et al. [40] characterized natural fibers extracted from the aninga (*Montrichardia linifera)* stem and developed a unidirectional polymeric sheet with an epoxy matrix. The results observed guarantee that the aninga fiber is considered to be of high performance (>200 MPa) and can be used in internal car components.

Owing to its large diversity of plants, the Amazon Forest has a great number of NLFs that are still little investigated [25,39]. This is the case with the fiber extracted from the plant popularly known as envira (*Bocageopsis multiflora*), common in the region that is found mainly in the states of Amazonas, Para, and Amapa in Brazil. From the stem of this plant, fibers are taken and used by indigenous tribes as well as small rural communities for the manufacture of ropes to tie huts and straw houses and to wrap manioc stalks, generating employment and income for these communities [41]. These uses indicate that envira fiber might have good mechanical properties, as well as a potential for reinforcement of polymer composites. Although the traditional uses of envira demonstrate promising properties, no studies have been found that present their mechanical properties and possible application as reinforcement in polymer composites. For the first time, a comprehensive investigation is conducted on the microstructure, thermal stability, and mechanical behavior of envira fibers. Furthermore, the interaction between envira fiber and the epoxy matrix in composites as well as its reinforcement potential. Specifically, the objective of this work was to investigate the envira fiber regarding its density, thermogravimetry behavior, morphology, and mechanical properties, as well as to evaluate the tensile and flexural properties of epoxy matrix composites incorporated with envira fiber.

## 2. Materials and Methods

### 2.1. Materials

Diglycidyl ether bisphenol-A (DGEBA) epoxy resin and triethylenetetramine (TETA) hardener, used in a ratio of 2 parts resin to 1 part hardener, were used as the matrix for the composite, supplied commercially by Epoxy Fiber (Sao Paulo, Brazil).

**Figure 1:**
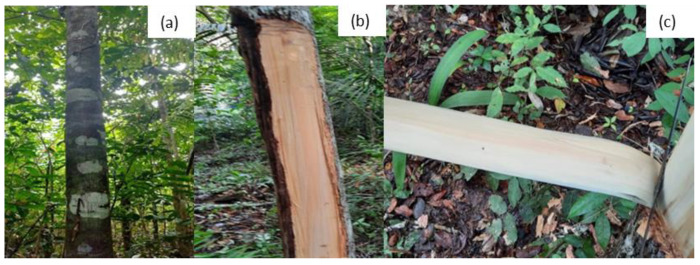
The envira: Envira plant (**a**), stem (**b**), and bark plant (**c**).

The envira fibers (*B. multiflora*) used in this research were supplied from local commerce in the city of Moju, Para, Brazil (01°53′10″ S and 48°46′00″ W). The fibers were extracted from the bark of the envira tree, which was manually extracted without harming or causing the death of the plant. After that, the fibers were washed, dried in a stove, manually separated into filaments, and cut into 300 mm lengths. Figure 1 shows the envira plant (a), the stem (b), and the bark (c).

### 2.2. Fiber Characterization

#### 2.2.1. Dimensional Characterization

The average diameter was measured using an Even optical microscope, model MDA 1300 (Future Win Joe, Xiasha, Hangzhou, China). Each fiber was measured at five different points, evenly separated, and then rotated by 90° for a new reading at the same five previous points. The length of each fiber was measured using a caliper with a precision of 0.01 mm, and its mass was weighed on a YMC electronic balance model Chyo JK-200 (Kyoto, Japan) with a precision of 0.0001 g. To calculate the volume (Vm), the fiber was considered to have a perfect cylindrical geometry, using:

(1) $$V_{m}=\frac{\pi d^{2}l}{4}$$

where Vm is average volume, d is diameter, and l is length.

From the values of average volume and mass (m), it was possible to calculate the density (“ρ”) using:

(2) $$\rho =\frac{m}{V_{m}\;}$$

#### 2.2.2. X-Ray Diffraction (XRD)

XRD was carried out on a Proto Manufacturing XRD Powder Diffraction System: 30 kV and 2 mA generator, Cu-Kα1 radiation, 0.0149° angular step, 0.5 s time interval, 47 min scan, and 2θ ranging from 5° to 60°. The calculation of the crystalline index (CI) followed the method described by Segal et al. [42], using the maximum intensity of the (1 0 1) and (0 0 2) peaks associated with the amorphous and crystalline phases of the fiber. The CI values were determined using Equation (3), where I1 is the intensity of the minimum (amorphous region) and I2 is the intensity of the diffraction maximum (crystalline region).

(3) $$\mathrm{CI}=\left(1-\frac{I_{1}}{I_{2}}\right)*100$$

The microfibril angle (MFA) was determined from the crystalline cellulose peak (0 0 2), according to the methodology described by Cave [43]. Based on XRD analysis, the MFA value is obtained from the relation between the Gauss curve and the first- and second-order derivative curves. From this analysis, the “T” parameter is obtained and applied to the polynomial equation:

MFA = − 12.19 ∗T3 + 113.67 ∗T2 − 348.40 ∗ T + 358.09(4)

### 2.3. Thermogravimetric Analysis (TGA)

TGA was performed in a simultaneous thermal analyzer, model STA 449 F3 Jupter da NETZSCH (Berlin, Germany), under a nitrogen atmosphere with a flow rate of 50 mL/min and a heating rate of 10 °C/min and a temperature range from 20 to 500 °C.

### 2.4. Fourier Transform Infrared Spectroscopy (FTIR)

The infrared spectra of the fiber samples and composites were obtained by attenuated total reflectance (ATR). The FTIR analysis was performed using a Bruker model Vertex 70v (Billerica, MA, USA), using a mid-infrared range (4000 cm^−1^–400 cm^−1^).

### 2.5. Mechanical Characterization of Fiber

One hundred envira fibers were individually tensile tested after being attached to a 90 m/g^2^ paper using epoxy adhesive. Each fiber tensile test was carried out according to the ASTM C1557 standard method [44], at room temperature (RT), using an Intermetric-iM50 universal testing machine (São Paulo, Brazil), with a 5 kN load cell and a speed of 0.5 mm/min for all tensile tests. The cross-section of the fibers was considered circular, and their cross-sectional areas were used to calculate the tensile strength. In addition, the deformation property was calculated from the load-displacement data, and the Young’s modulus was based on the slope of the stress-strain curve in the elastic region.

### 2.6. Preparation and Mechanical Characterization of Composites

Epoxy matrix composites for tensile and flexural testing were produced based on the standard methods ASTM D 638 [45], and ASTM D 790 [46]. Proportions of 10, 20, 30, and 40 vol% of envira fiber were used in production. The composites were produced by hand lay-up in a silicone mold. First, the epoxy resin was added to the mold, followed by the fibers. After adding the exact amount of fibers, the mold was then completely filled with resin. The specimens remained in the mold for 24 h, after which they were removed and sanded. Table 1 shows the nomenclature of the different composites.

The composites tensile tests were carried out at RT, according to the ASTM D 638 [45] using an Intermetric-iM50 (São Paulo, Brazil) universal testing machine, with a 5 kN load cell. The crosshead speed was set as 2 mm/min for all tensile tests. To calculate the strain property and Young’s modulus of the composites, the same calculation method used for the fibers was used.

The flexural tests were performed at RT using a three-point bending setup according to the ASTM D790 [46]. Specimens for each condition were tested using an Intermetric-iM50 (Sao Paulo, Brazil) universal machine, with a 5 kN load cell and 51.2 mm distance between supports.

Flexural strength Equation (5) and flexural modulus Equation (6) were then obtained using the expressions:

(5) $$\sigma_{f}=\;\frac{3PL}{2bd^{2}}$$

(6) $$E=\;\frac{L^{3}}{4bd^{3}}$$

where *P* is the load at a given point on the load deflection curve, *L* the support spain, *b* is the width of beam tested, *d* is the depth of beam tested, and *m* the slope of the tangent line.

### 2.7. Statistical Analysis

The statistical validation of the data was carried out using the analysis of variance (ANOVA), with a confidence of 95% (*p* < 0.05). The mean values were compared by the Tukey test.

### 2.8. Scanning Electron Microscopy (SEM)

The morphology of fibers and the fracture surface of composites were observed by SEM in a model Mira3 FEG 250 TESCAN microscope (Brno, Czech Republic) operating with secondary electrons at 5 kV and a work distance between 25 and 10 mm. The samples were first metallized with Au for 2 min, depositing a thin film on the surface of the sample.

## 3. Results and Discussion

### 3.1. Characterization of the Envira Fiber

**Figure 2:**
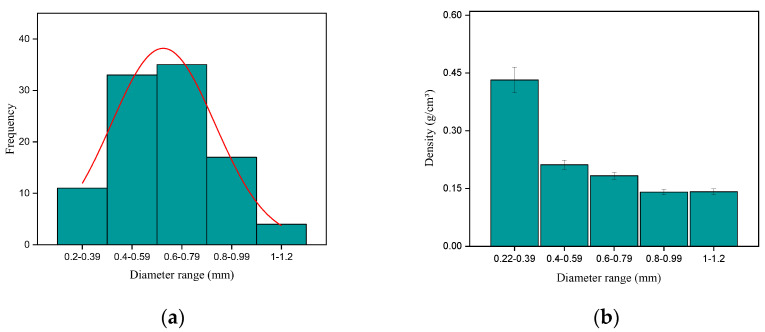
Dimensional characterization. (**a**) Frequency distribution pattern and (**b**) density distribution pattern of envira fibers.

Figure 2 shows the distribution of the number of envira fibers in relation to the diameter range (a) and the relation between density and diameter (b).

It is possible to observe an almost normal frequency pattern. It is also noted a greater amount of envira fibers in the diameter ranges of 0.40–0.59 mm and 0.60–0.79 mm. This result may be associated with the nature of the fibers that are generally more compact and smaller in diameter, and therefore less brittle [47,48,49]. Regarding density, there is a decrease in the average values with increasing diameter, a fact that may be associated with a greater amount of voids in the structure of larger diameter fibers. This same pattern was also observed for piassava fiber [47]. The average value obtained for this property was 0.23 g/cm^3^, which is lower when compared to densities of other Amazon NLFs [25,26,27,28,50,51]. According to [25], with the low value obtained for density, it is possible to produce lighter composites when compared to composites reinforced with synthetic fibers.

#### 3.1.1. XRD

**Figure 3:**
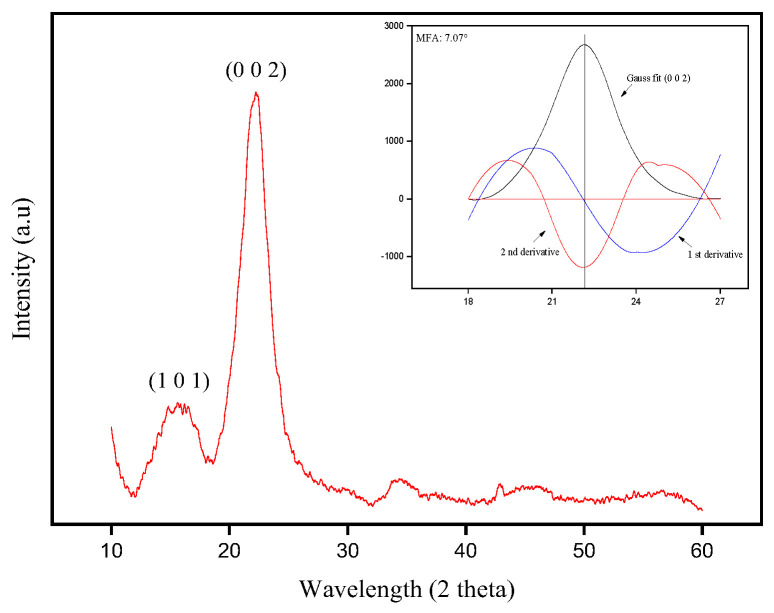
XRD pattern of the envira fiber.

Figure 3 shows the diffractogram obtained for the envira fiber.

A halo is observed at an angle of 16.5° and a peak at 22.7°, indicating the existence of amorphous constituents and crystalline cellulose in the fiber. The value of the CI obtained was 69.5%. This result is relatively close to those found by other authors for NLFs such as: ubim (63%) [26], malva (67.5%) [38], buriti (63.11%) [39], tucum (55.7%) [52], kenaf (44.3%) [53], banana (66.71%) [54], *Eulesine indica* (45%) [55], and guaruman (67%) [56]. The MFA obtained for envira fiber was 7.07°, which was calculated considering the cellulose crystalline peak at 22.5°. According to Segal [42], the MFA is related to the strength of the natural fiber. The value reported in this paper is comparable to the MFA of other NLFs [26,39,54,56].

#### 3.1.2. TGA Analysis

**Figure 4:**
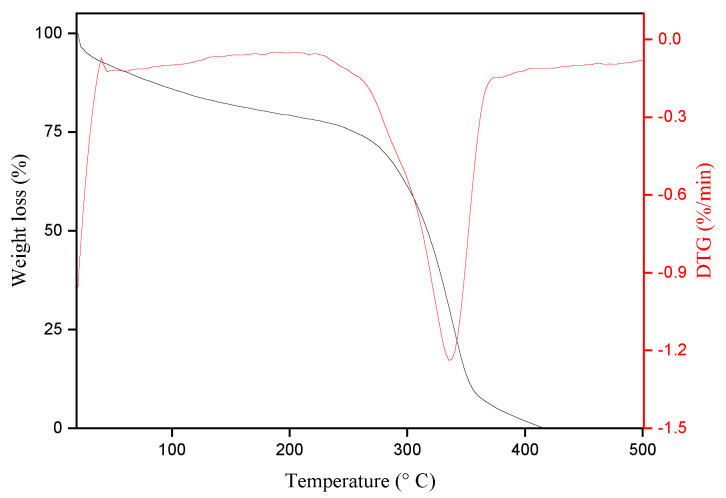
TGA/DTG curves obtained from the envira fibers.

TGA and its derivative (DTG) obtained for the envira fibers are shown in Figure 4.

It is possible to observe that envira fiber weight loss occurs within two temperature ranges. The first significant weight loss was observed at 45.3 °C with a weight reduction of approximately 9.5%. This weight loss may be related to the evaporation of the water present in the envira fiber. It is also observed that the fiber maintains thermal stability until 210 °C. The second weight loss of approximately 51% occurs between 250 and 350 °C, which may be related to thermal degradation of the cellulose. The TGA results of envira fiber indicate thermal behavior comparable to other NLFs that have been studied for possible applications in polymer composite materials [55,57,58,59,60,61,62]. According to Senthamaraikannan et al. [57], the evaluation of the thermal properties of these fibers is important to establish processing conditions that do not cause degradation and loss of property for these materials.

#### 3.1.3. FTIR Analysis

**Figure 5:**
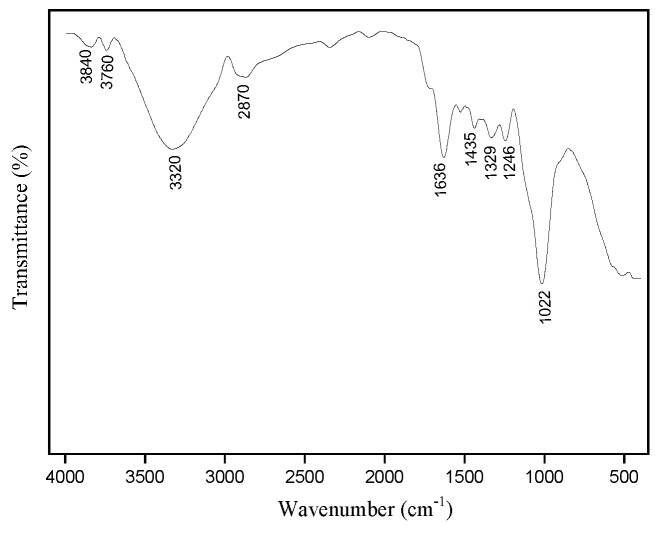
FTIR spectrum of the envira fiber.

Figure 5 shows the FTIR spectrum of envira fiber. Through this analysis it is possible to observe that envira fibers have vibrational properties similar to other NLFs [58,59,60,61,62,63,64,65,66,67,68,69,70]. According to Atangana et al. [68], NLFs are mainly composed of cellulose, hemicellulose, lignin, and waxes, which form in their structure alkanes, esters, aromatics, ketones, and alcohols, being associated with a functional group responsible for a type of vibration at a specific wave number.

Through the FTIR spectrum of envira fiber, it is possible to observe the existence of main bands between 3100 cm^−1^ and 3900 cm^−1^ that are attributed to the elongation of the OH group, evidencing the presence of cellulose and lignin [65,68,70,71,72]. The band at 2870 cm^−1^ can be attributed to CH_2_ groups, observed in cellulose and hemicellulose molecules [56,62,67,69,71]. The band 1630 cm^−1^ may be associated with the elongation of C = O present in lignin and hemicellulose [56,68,73]. At 1435 cm^−1^ symmetric bending of CH_2_ is observed, denoting the presence of cellulose [68,73]. The band at 1329 cm^−1^ refers to deformation vibrations of the OH bond in alcohols present in the cellulose constitution [55,66]. At 1246 cm^−1^ stretching vibration of the acetyl group in lignin [70]. The band around 1022 denotes the presence of lignin due to the elongation of C-OH [39,68].

#### 3.1.4. Analysis of the Morphology of Envira Fiber

**Figure 6:**
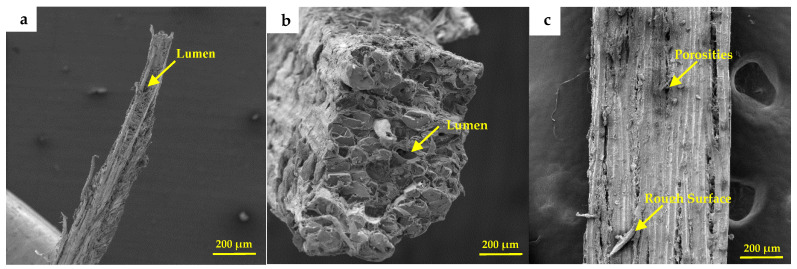
Scanning electron microscopy of the envira fiber: (**a**) cross-section at 200× magnification, (**b**) cross-section at 1000× magnification, and (**c**) longitudinal section.

The morphology of the envira fiber analyzed by SEM is shown in Figure 6.

In Figure 6a, it is possible to note that the envira fiber has an irregular geometry, composed of thin bundles of fibrils. In addition, it presents flaws and irregularities along its length. In Figure 6b the micrography shows the fiber cross section, where one can observe the presence of lumens, which are channels responsible for nutrient transfer along the fiber structure [39,68]. It is also observed in Figure 6c that the fiber has a rough surface and the presence of porosity. According to literature [67,72,74,75,76,77] the presence of pores in the fibrillar structure can affect the fiber strength properties; however, they can help in better interfacial bonding between matrix and fiber. The morphological aspects presented for envira fiber are similar to other studies performed for different NLFs [39,55,56,66].

#### 3.1.5. Mechanical Characterization of the Envira Fiber

**Figure 7:**
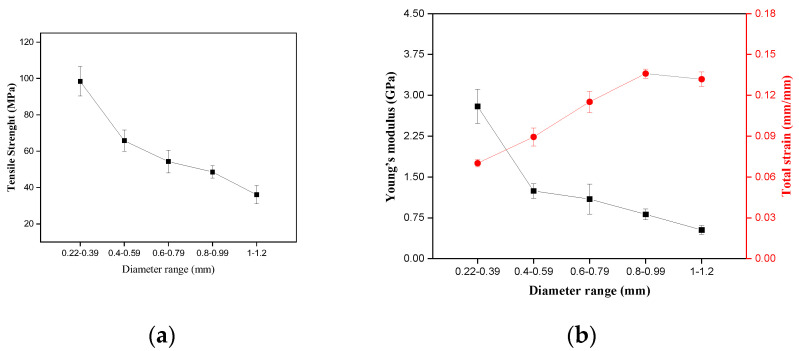
Tensile strength of envira fiber (**a**), Young’s modulus, and strain (**b**).

Figure 7a shows the tensile strength results, and Figure 7b shows the results for Young’s modulus and strain in relation to the envira fiber diameter.

Regarding the fiber tensile strength, Figure 7a, there is a decrease in the average values with the increase of the diameter interval. This pattern may be related to a larger amount of defects in the fiber structure with larger diameters. Studies with different NLFs show the same pattern observed for envira fiber [47,74,75]. According to Ferreira et al. [47], the less thick fibers tend to be more uniform and present in their structure more compact microfibrils and fewer voids, and thus present higher strength when compared to larger diameter fibers.

Regarding Young’s modulus, in Figure 7b, it is observed that its average value decreases with increasing diameter range, indicating that larger diameter fibers are less stiff when compared to thinner fibers. This pattern can be attributed to microstructural aspects, such as the presence of roughness and porosity in the fiber [47,48,49]. For strain, Figure 7b, an almost directly proportional relationship of this property is observed with the increase in fibrillar diameter, confirming the fiber stiffness results. The statistical analysis of the mechanical characterization of the fiber is presented in Table 2.

The ANOVA values for strength show that the calculated F (19.661) is higher than the critical F value (2.467). For Young’s modulus, the calculated F (18.690) is higher than the critical F value (2.467), and for total strain, the calculated F (26.224) is also higher than the critical F value (2.479). Thus, based on these results, the hypothesis that the means of the properties presented are equal is rejected, with a confidence level of 95%. Based on the ANOVA results, Tukey’s test is necessary to investigate whether the increase in envira fiber diameter causes significant changes in the fiber’s mechanical properties. Table 3 shows the results of Tukey’s test.

The minimum significant difference (m.s.d) is a value that indicates which treatment differs in its mean values. When the absolute value of the difference between two means is equal to or greater than the m.s.d value, the means are considered statistically different. The value of m.s.d for strength was calculated as 31.820; for Young’s modulus the m.s.d was calculated as 1.054, and for total strain the m.s.d was 0.027. Thus, it is evident that the diameter causes changes in the mechanical properties of the fiber. The smaller diameter intervals present, in fact, the highest tensile strengths and Young’s modulus when compared to the larger diameters. Regarding the strain, the highest values are related to the thicker fibers, suggesting that these fibers may be more ductile than the smaller diameter fibers.

### 3.2. Characterization of Composite Materials

#### 3.2.1. Characterization of Epoxy Matrix Composites by FTIR

**Figure 8:**
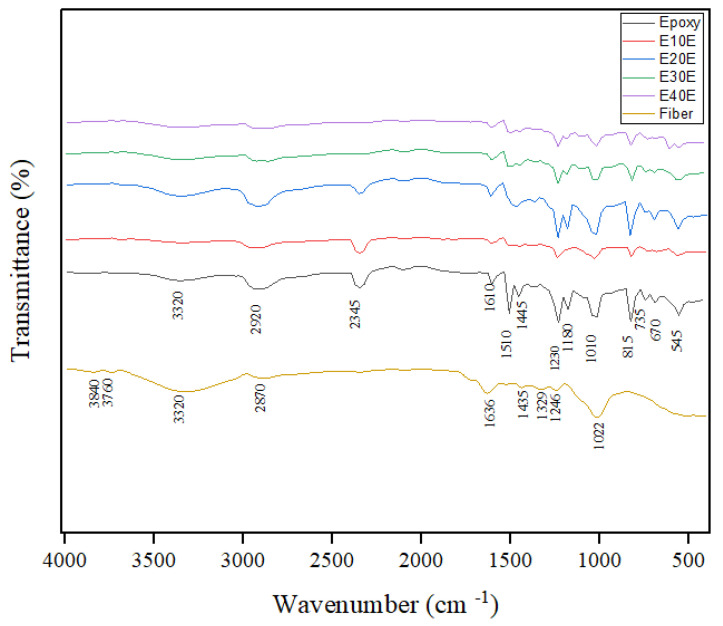
FTIR graph obtained for epoxy resin, composites, and fiber.

Figure 8 shows the curves obtained from the FTIR analysis of the composites.

Various studies on the incorporation of NLFs into an epoxy matrix have shown an FTIR spectrum similar to that observed in this study [21,61,62,63,64,65]. Neves et al. [76] and Gargol et al. [77] observed that the bands found refer predominantly to the spectrum of the epoxy resin. In fact, when compared to the spectra of envira fibers, the bands referring to the lignocellulosic components are observed to be less intense. The band at 3320 cm^−1^ is related to the stretching of the bonds of the OH group of cellulose. According to Kumaar et al. [78] the appearance of this band indicates interactions between the composites and the epoxy resin. The band at 2940 cm^−1^ can be attributed to the symmetrical stretching vibration in the single bond of the CH_3_ group of the epoxy resin [79,80]. Around 1710 cm^−1^ a band that corresponds to the C=O stretching vibration of the carboxylic acid in the lignin or ester components in the fiber hemicellulose [77]. The bands at 1610 and 1560 cm^−1^ may be associated with the C=C vibrations of the aromatic ring in the epoxy resin [76,80]. At 1445 cm^−1^ and 1230 cm^−1^ they are attributed to CH_2_ deformation [81]. The bands at 1180 and 1010 cm^−1^ indicate the vibration of the CO bond of the ether in the resin [31,82]. A band at 815 cm^−1^ is attributed to the vibration of the epoxy group [76]. The 1940, 1510, 1180, and 1010 cm^−1^ bands, which also refer to the characteristic groups present in lignocellulosic fibers, are present in the composite spectra, but to a lesser extent. According to Neves et al. [76] this factor can be interpreted as an indication of active interaction between the molecular groups of the epoxy and the fibers. Moreover, it is possible to observe that the bands characteristic of the presence of cellulose, the main component of lignocellulosic fibers, shown in Figure 5, are not observed in the FTIR spectra of the composites with added fibers, as indicated by the arrow in the band around 3700, a fact that may be related to the higher amount of resin in relation to the envira fiber in the composites.

#### 3.2.2. Tensile Mechanical Properties

**Figure 9:**
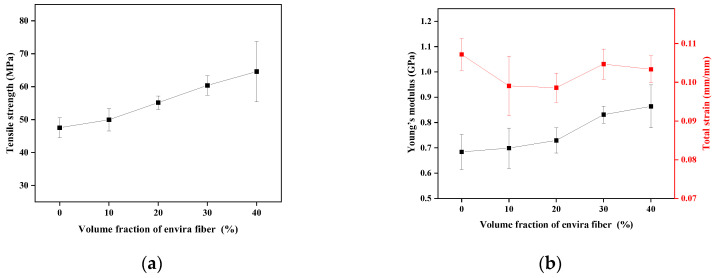
Graphs of the tensile mechanical properties of composites: tensile strength (**a**) and Young’s modulus and strain (**b**).

The tensile properties of the epoxy matrix composite, with variation in fiber percentage, are shown in Figure 9.

When observing the average strength values, the composite with 40% by volume of envira fiber showed the best result. Additionally, Figure 9 demonstrates that the tensile strength of the composites exhibited a trend of linear growth. According to Abd El-Bak et al. [83], the relative quantities of fibers have noticeable effects on mechanical properties; the author notes that tensile strength increases as the amount of fiber increases. However, the composition with the highest fiber concentration showed a significant dispersion in its results. This may be related to the large amount of fiber in the matrix, which could have affected the fiber’s wettability, complicated the production of the composites, and interfered with the adhesion of the fiber to the matrix. The strength values obtained indicate that envira fiber exhibits similar or superior properties compared to other lignocellulosic fibers [25,30,50].

In their study with long tucum fibers, Oliveira et al. [25] obtained a strength result of 38.8 MPa, which is lower than the value observed in this study for the same amount of fiber. In a study with piassava fibers, Castro et al. [50] obtained a tensile strength value of 15 MPa with 30% by volume of fiber of 30 mm, a value much lower than that found in the present study, a fact that may be related to the length of the fiber; the author identified a decrease in relation to the matrix. Similar to the present work, the results showed a trend of linear growth in the average strength values. However, these values are lower when compared to the average strength value for the composition with 30% by volume of envira fiber.

Regarding the Young’s modulus, the same pattern observed for strength is noted, indicating that the stiffness of the composite increases as fibers are added to the matrix. In contrast, the deformation results did not show a linear trend; the results indicate that the addition of envira fibers in epoxy decreases the values for this property. The analysis of variance obtained for the tensile properties is presented in Table 4.

The ANOVA values for strength show that the calculated F (12.781) is higher than the critical F value (2.75). For modulus of elasticity, the calculated F (9.018) is higher than the critical F value (2.75), and for total strain, the calculated F (3.492) is also higher than the critical F value (2.75). Thus, based on these results, the hypothesis that the means of the properties presented are equal is rejected, with a confidence level of 95%. Based on the ANOVA results, Tukey’s test is necessary to investigate whether the increase in envira fiber volume causes significant changes in the composites’ mechanical properties. Table 5 shows the results of Tukey’s test.

The m.s.d for tensile strength was calculated as 8.222. For the Young’s modulus, the m.s.d was calculated as 0.112, and for the total deformation, the m.s.d was 0.008. Thus, it was possible to observe that the mechanical properties of the epoxy-envira composites are influenced by the fiber concentration. According to the Tukey test results, the addition of 30% and 40% by volume differs statistically from the values of the other compositions regarding strength and modulus. In terms of deformation, only the composition with 20% by volume of fiber shows a difference compared to the matrix. Therefore, it can be concluded that the fiber volume induces changes in the mechanical properties of the envira fiber-reinforced composites, with the composition containing the highest fiber volume demonstrating the best strength results.

##### Microstructural Analysis of Fractured Surface

**Figure 10:**
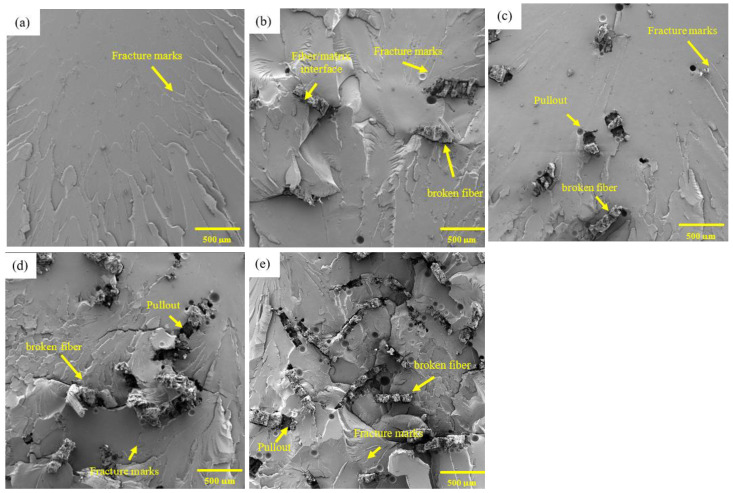
Analysis of the fracture region of the epoxy resin (**a**) and the composites with 10 (**b**), 20 (**c**), 30 (**d**), and 40 (**e**) vol% of envira fiber, tested in tensile strength.

Micrographs obtained by SEM of the fracture region of the composites reinforced with envira fibers are shown in Figure 10.

It can be observed in the SEM images that both the micrograph of the fracture region of the epoxy resin, Figure 10a, and those of the envira fiber-reinforced composites, Figure 10b–e, exhibit delamination and fracture fringes, which are typical characteristics of brittle fracture in polymers. Additionally, in the compositions with fiber addition, there is partial adherence of the fibers to the matrix, fiber pull-out, and pores, which are aspects that may undermine the reinforcement of the composites and reduce the material’s strength [56]. Various studies have reported that a chemical treatment on the fibers can improve fiber adhesion, improving the strength of the fibers [84,85,86,87].

Furthermore, the presence of broken envira fibers in the composites with 30 and 40 vol% of fiber suggests good adhesion of the fibers to the matrix. According to Oliveira et al. [25], broken fibers indicate that a reasonable load transfer occurred between the fiber and the matrix; when this transfer does not happen effectively, a decrease in the strength of the composite occurs. In these compositions, it is also observed that the fibers act as barriers against cracks propagating through the epoxy matrix, and this barrier can contribute to the reinforcement of the composite [56]. The patterns observed in these micrographs confirm the results obtained for the mechanical strength property.

In Figure 10e a high concentration of defects, such as bubbles and pores, can be observed, which may be due to the difficulty in molding with a high concentration of envira fibers. This pattern may have influenced the wide dispersion of results in the composition with 40% fiber, as identified in Figure 9a.

#### 3.2.3. Flexural Strength

**Figure 11:**
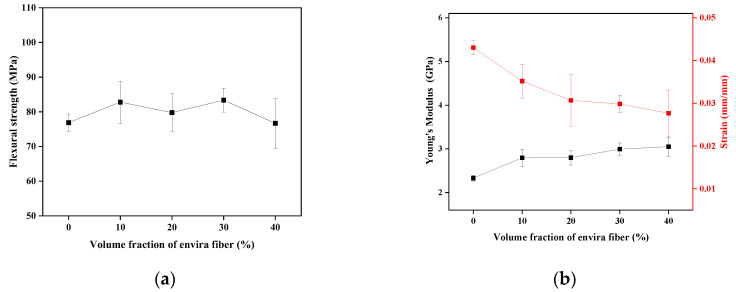
Flexural properties of the composites analyzed. (**a**) the flexural strength and in (**b**) the flexural modulus and deformation.

The mechanical properties obtained in flexural are presented in Figure 11.

The results indicate that the addition of envira fiber has no apparent influence on the flexural strength results. The highest average value is related to the composition with 30 vol% fibers; nevertheless, the standard deviation bars indicate that there is not a significant difference between the results. Furthermore, the irregular behavior of the strength values may be related to flaws in the manual production process of the composites, causing the presence of bubbles, which may have acted as stress concentrators, weakening the material. Aruchamy et al. [88] observed this same pattern where higher concentrations of fibers decrease the composite’s strength due to the low adhesion between the fiber and the matrix.

For the flexural modulus, the average values increase with the insertion of fibers into the matrix. The composition with 40 vol% of envira fibers showed the highest average value for the bending modulus, with a 30 vol% increase compared to the epoxy matrix. Zhang et al. [80], when evaluating the flexural properties of epoxy matrix composites reinforced with bamboo fibers, observed this same pattern. According to the authors, the addition of NLFs to epoxy provides greater bending stiffness to the composites during the bending test due to the better mechanical properties, such as modulus, stiffness, and flexibility of the fibers, when compared to the epoxy resin. Regarding deformation, there is a decrease in the average value, with the highest value associated with the epoxy resin. This value decreases with the insertion of fibers into the matrix, a result that supports the findings for the modulus of elasticity, as the greater the volume of fibers in the matrix, the greater the stiffness of the composite. Although no statistically significant variation was observed in flexural strength, it can be inferred that the incorporation of fibers led to an increase in the average values of this property, particularly with the addition of 30 vol%. This suggests that the composite may be suitable for applications involving flexural mechanical loading. However, fiber contents exceeding 30 vol% appear to saturate the matrix, resulting in a decrease in mechanical strength.

Statistical analysis of flexural properties is presented in Table 6.

The ANOVA results indicated a significant difference in the modulus and strain results. Table 7 presents the values of the differences between the means after the Tukey test.

The obtained m.s.d values for strength and bending modulus were 0.279 and 0.007, respectively. According to the Tukey test results for the Young’s modulus and strain, only the value for the epoxy resin shows a statistical difference from the other compositions, confirming the results observed in Figure 11.

##### Microstructural Analysis of Flexural Fractures

**Figure 12:**
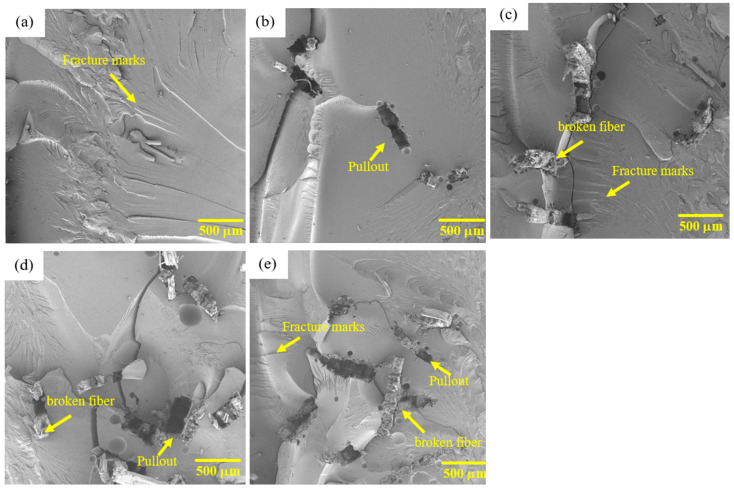
Analysis of the fracture region of epoxy resin (**a**) and composites with 10 (**b**), 20 (**c**), 30 (**d**), and 40 (**e**) vol% envira fiber after flexural strength testing.

Figure 12 presents the fracture region of the composites tested in flexural.

It can be observed in the SEM images of the composites tested in flexural that both the micrograph of the fracture region of the epoxy resin, Figure 12a, and the composites reinforced with envira fibers, Figure 12b–e, exhibit the same pattern observed for the compositions tested in tensile, with the presence of delamination and fracture fringes. As previously indicated, these are typical characteristics of brittle fracture exhibited by polymers. It is also noted that in the compositions with fiber addition, there is partial adhesion of the fibers in the matrix, pulled-out fibers, the presence of bubbles, and broken fibers. Additionally, a large number of pores are evident, which may have acted as stress concentrators, explaining the results presented for flexural strength. According to Aruchamy et al. [70], who observed these characteristics in their composites when tested in flexural, the bubbles and pulled-out fibers indicate an inadequate relationship between the fiber and the matrix, reducing the flexural strength of the composites.

The results obtained in this work indicate that envira fiber has great potential for use as a reinforcement in polymer composites, since it has favorable characteristics for this use. The density of the fiber is one of the lowest observed in the literature for NLFs, which can contribute to the production of lightweight materials. The tensile and flexural strengths show interesting values, which can contribute to the production of less expensive materials when compared to materials that use reinforcements derived from fossil fuels.

## 4. Conclusions

The fiber density measured by the geometric method was 0.23 g/cm^3^. The XRD result indicated a value of 69.5% for the crystallinity index and 7.07° for the MFA. The thermogravimetry result indicated thermal stability of envira fiber around 210 °C. FTIR indicated the presence of different functional groups found in cellulose, hemicellulose, lignin, and waxes, the main components of lignocellulosic fibers. Analysis of the fiber morphology revealed the presence of fibrils, defects, and microstructural irregularities along its length. The mechanical characterization of the fiber by tensile test indicated a decrease in strength with increasing fiber diameter, a pattern already expected for lignocellulosic fibers.

The incorporation of envira fiber into the epoxy matrix resulted in an increase in tensile strength and led to a slight enhancement in the composite’s stiffness. Although the mean values indicated superior strength for the formulation containing 40 vol% fiber, statistical analysis suggests that the optimal composition is 30 vol%, which exhibited a 27% increase in tensile strength compared to the plain epoxy matrix. The analysis of the fracture region of the composites revealed the presence of delamination and areas where the fibers were pulled out of the matrix, indicating weak interfacial adhesion. However, the compositions with larger amounts of fibers showed a significant number of broken fibers, which may influence the improvement in the strength of these composites. These results indicate the great potential of these fibers, since they can replace their synthetic counterparts, such as carbon and glass fibers, contributing to the production of less expensive, lighter, and environmentally friendly materials.

In relation to the properties of the composites tested in flexural strength, the addition of envira fibers did not influence the strength results. This may be related to the manual manufacturing method, which can lead to the presence of bubbles that weaken the material.

The incorporation of envira fiber in an epoxy matrix showed to be efficient, considering that the produced composites obtained a tensile strength value higher than NLFs composites researched in the literature. Furthermore, envira fiber is for the first time investigated for application in composites; it is a relatively unknown natural fiber from the northern region of Brazil, of low cost, and that has shown promising results regarding its use as reinforcement in polymeric composites. These new composites can be applied as substitutes for conventional composites reinforced with synthetic fibers. Moreover, as it is a lighter material, it can be applied in civil construction by replacing high performance wood.

## Figures and Tables

**Table 1:** Classification and nomenclature of composite materials.

Raw Materials	Compositions				
	E0E	E10E	E20E	E30E	E40E
Fiber (%)	0	10	20	30	40
Resin (%)	100	90	80	70	60
Number of samples	8	8	8	8	8

**Table 2:** Analysis of variance (ANOVA) of tensile strength of envira fiber.

**Maximum Strength (MPa)**						
**Source**	**Sum of Squares**	**Degrees of Freedom**	**Mean of Squares**	**F (Calculated)**	***p*** **-Value**	**F Critical**
Between the groups	59,770.61	4	14,942.65	19.661	8.14 × 10^−12^	2.467
Inside the group	72,201.27	95	760.013			
Total	13,1971.9	99				
**Young’s Modulus (GPa)**						
**Source**	**Sum of Squares**	**Degrees of Freedom**	**Mean of Squares**	**F (Calculated)**	***p*** **-Value**	**F Critical**
Between the groups	62.30349	4	15.57587	18.690	2.32 × 10^−11^	2.467
Inside the group	79.17237	95	0.833393			
Total	141.4759	99				
**Total Strain (mm/mm)**						
**Source**	**Sum of Squares**	**Degrees of Freedom**	**Mean of Squares**	**F (Calculated)**	***p*** **-Value**	**F Critical**
Between the groups	0.057186	4	0.014296	26.2243	3.56 × 10^−14^	2.479
Inside the group	0.046338	85	0.000545			
Total	0.103524	89				

**Table 3:** Differences between the average values of the diameter intervals after applying the Tukey test.

Tensile Strength (m.s.d = 31.820)						Young’s Modulus (m.s.d = 1.054)					Total Strain (m.s.d = 0.027)				
	0.22–0.39	0.40–0.59	0.60–0.79	0.80–0.99	1.00–1.20	0.22–0.39	0.40–0.59	0.60–0.79	0.80–0.99	1.00–1.20	0.22–0.39	0.40–0.59	0.60–0.79	0.80–0.99	1.00–1.20
0.22–0.39	0	**41.685**	**53.190**	**58.862**	**71.424**	0	**1.553**	**1.701**	**1.982**	**2.268**	0	0.019	**0.045**	**0.066**	**0.062**
0.40–0.59	**41.685**	0	11.506	17.178	29.740	**1.553**	0	0.148	0.429	0.715	0.019	0	0.026	**0.047**	**0.042**
0.60–0.79	**53.190**	11.506	0	5.672	18.234	**1.701**	0.148	0	0.280	0.567	**0.045**	0.026	0	0.021	0.017
0.80–0.99	**58.862**	17.178	5.672	0	12.562	**1.982**	0.429	0.280	0	0.287	**0.066**	**0.047**	0.021	0	0.004
1.00–1.20	**71.424**	29.740	18.234	12.562	0	**2.268**	0.715	0.567	0.287	0	**0.062**	**0.042**	0.017	0.004	0

The significant differences are highlighted in bold.

**Table 4:** Analysis of variance (ANOVA) of the tensile strength of envira fiber reinforced composites.

**Maximum Strength (MPa)**						
**Source**	**Sum of Squares**	**Degrees of Freedom**	**Mean of Squares**	**F (Calculated)**	***p*** **-Value**	**F Critical**
Between the groups	1203.898	4	300.974	12.781	8.48 × 10^−6^	2.75
Inside the group	588.726	25	23.549			
Total	1792.624	29				
**Young’s Modulus (GPa)**						
**Source**	**Sum of Squares**	**Degrees of Freedom**	**Mean of Squares**	**F (Calculated)**	***p*** **-Value**	**F Critical**
Between the groups	0.157	4	0.039	9.018	1.19 × 10^−4^	2.75
Inside the group	0.109	25	0.004			
Total	0.267	29				
**Total Strain (mm/mm)**						
**Source**	**Sum of Squares**	**Degrees of Freedom**	**Mean of Squares**	**F (Calculated)**	***p*** **-Value**	**F Critical**
Between the groups	0.000328	4	8.19 × 10^−5^	3.492	0.021	2.75
Inside the group	0.000587	25	235 × 10^−5^			
Total	0.000914	29				

**Table 5:** Results obtained for differences between mean values after applying Tukey’s test.

Maximum Strength (m.s.d = 8.222)						Young’s Modulus (m.s.d = 0.112)					Total Strain (m.s.d = 0.008)				
	E0E	E10E	E20E	E30E	E40E	E0E	E10E	E20E	E30E	E40E	E0E	E10E	E20E	E30E	E40E
E0E	0	2.420	7.601	**12.837**	**17.053**	0	0.015	0.045	**0.147**	**0.18**	0	0.007	**0.009**	0.002	0.004
E10E	2.420	0	5.182	**10.417**	**14.633**	0.015	0	0.030	**0.132**	**0.165**	0.007	0	0.001	0.006	0.004
E20E	7.601	5.182	0	5.235	**9.452**	0.045	0.030	0	0.101	**0.134**	**0.009**	0.001	0	0.006	0.005
E30E	**12.837**	**10.417**	5.235	0	4.217	**0.147**	**0.132**	0.101	0	0.033	0.002	0.006	0.006	0	0.001
E40E	**17.053**	**14.633**	**9.452**	4.217	0	**0.18**	**0.165**	**0.134**	0.033	0	0.004	0.004	0.005	0.001	0

The significant differences are highlighted in bold.

**Table 6:** Analysis of variance (ANOVA) of the flexural properties of Envira fiber-reinforced composites.

**Flexural Strength (MPa)**						
**Source**	**Sum of Squares**	**Degrees of Freedom**	**Mean of Squares**	**F (Calculated)**	***p*** **-value**	**F Critical**
Between the groups	239.582	4	59.895	2.205	0.097	2.75
Inside the group	678.893	25	27.155			
Total	918.475	29				
**Young’s Modulus (GPa)**						
**Source**	**Sum of Squares**	**Degrees of Freedom**	**Mean of Squares**	**F (calculated)**	***p*** **-value**	**F critical**
Between the groups	1.921	4	0.480	17.654	5.3 × 10^−7^	2.75
Inside the group	0.680	25	0.027			
Total	2.601	29				
**Total Strain (mm/mm)**						
**Source**	**Sum of Squares**	**Degrees of Freedom**	**Mean of Squares**	**F (calculated)**	***p*** **-value**	**F critical**
Between the groups	0.00089	4	0.000222	12.741	8.69 × 10^−6^	2.75
Inside the group	0.00043	25	1.75 × 10^−5^			
Total	0.00132	29				

**Table 7:** Results obtained for differences between mean values of the flexural strength after applying Tukey’s test.

Young’s Modulus (m.s.d = 0.279)						Total Strain (m.s.d = 0.007)				
	E0E	E10E	E20E	E30E	E40E	E0E	E10E	E20E	E30E	E40E
E0E	0	**0.465**	**0.468**	**0.664**	**0.719**	0	**0.008**	**0.012**	**0.013**	**0.015**
E10E	**0.465**	0	0.002	0.198	0.254	**0.008**	0	0.004	0.005	0.007
E20E	**0.468**	0.002	0	0.196	0.251	**0.012**	0.004	0	0.001	0.003
E30E	**0.664**	0.198	0.196	0	0.055	**0.013**	0.005	0.001	0	0.002
E40E	**0.719**	0.254	0.251	0.055	0	**0.015**	0.007	0.003	0.002	0

The significant differences are highlighted in bold.

## Data Availability

The original contributions presented in this study are included in the article. Further inquiries can be directed to the corresponding author.
